# Epigenetic Regulation of Monoallelic Rearrangement (Allelic Exclusion) of Antigen Receptor Genes

**DOI:** 10.3389/fimmu.2014.00625

**Published:** 2014-12-05

**Authors:** Rena Levin-Klein, Yehudit Bergman

**Affiliations:** ^1^Department of Developmental Biology and Cancer Research, Institute for Medical Research Israel Canada, Hebrew University Medical School, Jerusalem, Israel

**Keywords:** asynchronous replication, immunoglobulin, V(D)J recombination, DNA methylation, hematopoietic development

## Abstract

While most genes in the mammalian genome are transcribed from both parental chromosomes in cells where they are expressed, approximately 10% of genes are expressed monoallelically, so that any given cell will express either the paternal or maternal allele, but not both. The antigen receptor genes in B and T cells are well-studied examples of a gene family, which is expressed in a monoallelic manner, in a process coined “allelic exclusion.” During lymphocyte development, only one allele of each antigen receptor undergoes V(D)J rearrangement at a time, and once productive rearrangement is sensed, rearrangement of the second allele is prevented. In this mini review, we discuss the epigenetic processes, including asynchronous replication, nuclear localization, chromatin condensation, histone modifications, and DNA methylation, which appear to regulate the primary rearrangement of a single allele, while blocking the rearrangement of the second allele.

## Introduction

In diploid genomes, most genes are transcribed and expressed from both maternal and paternal alleles, giving rise to a robust expression pattern, which allows the cells to be less susceptible to the damaging consequences of mutations and varying environmental cues. There is, however, a subset of genes that are expressed from only a single allele in a given cell, seemingly sacrificing the evolutionary benefits of the diploid genome (1). This apparent disadvantage is compensated for by other benefits, such as greater cell to cell diversity, which may allow greater robustness of the organism as a whole.

Monoallelic expression can be divided into two subgroups, non-random and random monoallelically expressed (RME) genes. The imprinted genes are a well-studied example of the non-random monoallelically expressed subgroup. In this subgroup, each gene is predetermined to be expressed either exclusively from the maternal or the paternal allele in all cells in the organism (2). Most monoallelically expressed genes fall into the second category of the RME genes. Here, each cell may express either the maternal or paternal allele, and the choice of which allele of each gene is expressed varies throughout the different cells in the body. One well-studied example of RME genes is the group of X chromosome linked genes in female mammals, where in each cell most of the genes on a single X chromosome are silenced epigenetically to create a balanced level of expression relative to that in male cells (3). Olfactory receptor genes constitute an additional family of well-defined RME genes (4). In each olfactory neuron, a single olfactory receptor is expressed from a single allele, to enable varied and specific odorant sensing. Recent studies have demonstrated that monoallelic expression is widespread in various tissues and that the percent of monoallelic expression rises sharply following differentiation from the pluripotent state (5–7). The families of genes that are subject to RME are numerous and vary highly (5–8).

## Allelic Exclusion of Antigen Receptor Genes

Historically, one of the earliest monoallelically expressed gene families to be recognized is that of the antigen receptors (9, 10). Each B and T cell recognizes only one antigen, as a result of the expression of a single functional V(D)J rearranged protein for each subunit of the antigen receptors. Expression of additional functional rearranged subunits from the second allele could lead to multiple specificities, with deleterious results such as autoimmunity (11). The phenomenon of monoallelic rearrangement of the antigen receptors has been coined “allelic exclusion.” During B and T cell development, at the proper developmental stage, each antigen receptor locus becomes accessible to the rearrangement machinery, and one of the two alleles undergoes rearrangement. If this rearrangement produces a functional antigen receptor chain, further rearrangements are prevented by a feedback inhibition mechanism. If, however, the rearrangement fails to produce a functional protein, further rearrangements on the original allele, or on the other allele are induced until a functional protein is produced (12, 13). The only antigen receptor locus that is not subject to feedback inhibition, is the TCRα locus, where rearrangements on both alleles are seen in most mature T cells (14).

Monoallelic expression may be regulated at multiple levels. Most RME genes are regulated at the transcriptional level, so that only one allele has the possibility of being transcribed. In contrast, in the case of allelic exclusion at the antigen receptor loci, both alleles (functionally and non-functionally rearranged) may be transcribed. However, only one of these alleles will give rise to a functionally translated protein. In fact, in an engineered mice where both endogenous alleles of either the IgH (15) or Igκ (16) loci are functionally prerearranged (i.e., the Ig alleles are both in a rearranged form prior to the cell stage when rearrangement usually occurs), both alleles are transcribed and translated in mature B cells. This signifies that the mechanisms ensuring monoallelic expression at these loci are not regulating transcription, but rather the rearrangement process itself.

There is a large amount of evidence at the Igκ locus that the primary allele which undergoes rearrangement is determined prior to the developmental stage when the rearrangement itself occurs. At an early developmental stage, the allele is selected randomly, so that overall in the B cell pool both alleles are represented equally. Only later in B cell development, does this choice become stable and clonally maintained in an epigenetic manner. Lineage tracing experiments in mice where the two Igκ alleles could be differentiated by flow cytometry showed that at early stages of B cell development, namely in hematopoietic stem cells (HSCs) and multipotent progenitor (MPP) cells, the choice of which allele is selected for rearrangement is still plastic (17). Thus, mature B cells arising from a single MPP or HSC will express either one of the Igκ alleles. At the CLP stage, however, following commitment to the lymphoid lineage, but prior to B cell commitment, two subgroups are observed. The first subgroup behaves like the earlier stages of development, where both alleles are represented on the surface of different mature B cells arising from a single CLP. The second subgroup of CLPs, however, shows a committed phenotype, where all of the mature B cells that originated from a single CLP express Igκ chains from the same allele (17). This commitment is observed in all stages following the CLP stage (pro-B and pre-B cells) leading up to the rearrangement process (17). It is plausible to assume that the committed CLP subgroup is at a later developmental stage than the uncommitted CLP subgroup, though they both fall under the definition of CLP cells. This developmental transition from a plastic to committed allelic phenotype seen in hematopoietic development strikes a delicate balance between the need for diversity in the organism, which is made possible by the early, non-committed stages, and the necessity to ensure monoallelic rearrangement at the proper stage. It will be interesting to investigate the precise developmental allelic commitment of other RME genes, including additional antigen receptor loci.

Interestingly, the choice made at the CLP stage is strong enough to override the feedback inhibition, as seen in the case of mice, which have one functionally prerearranged Igκ allele, and the other allele is in the germline configuration (16). In these mice, approximately 50% of the mature B cells express only the prerearranged allele, while the rest of the cells express both the prerearranged and a newly rearranged allele, signifying that the transcription of the prerearranged allele is not sufficient to supersede the cells where the germline allele was “chosen” for rearrangement.

## Asynchronous Replication

There are a number of epigenetic mechanisms that appear to regulate the selection of a single allele for rearrangement, and allow the recombination machinery to access only the chosen allele, while repressing the other allele and making it inaccessible. One of the mechanisms that mark the antigen receptor loci already at an extremely early developmental stage is the asynchronous replication timing of the DNA during S phase of the cell cycle.

Replication timing of DNA has been seen to be mostly in correlation with the expression patterns of the genes located within the region of replication (18, 19). Early replicating zones contain a large proportion of genes that are actively expressed, while repressed genes are mostly associated with late replicating zones. Approximately 10% of the regions in the genome fall into a third category of replication timing, where one of the alleles replicates early in S phase, while the replication of the second allele takes place only later (20). Genes located within asynchronously replicating regions are often expressed monoallelically. Most well-known examples of genes expressed monoallelically, such as imprinted genes (21), olfactory receptor genes (22), and genes silenced monoallelicaly on the X chromosome in females (23) are replicated in such a manner. The replication patterns are established in the early embryo, following implantation, and regions that replicate asynchronously starting from this stage remain so throughout development (24, 25). The asynchronous replication pattern remains even in cell types where the genes contained within the replication zone are not expressed (24). Thus, asynchronous replication is an early epigenetic mark of the potential of monoallelic expression. This potential is not necessarily realized in all cells.

**Figure 1:**
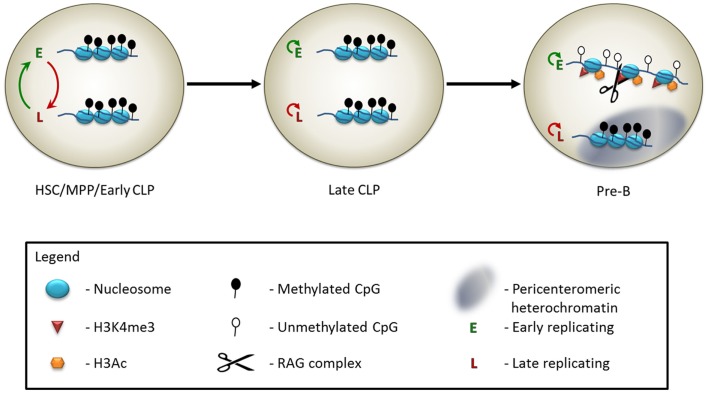
**Model of the epigenetic control of Igκ monoallelic rearrangement**. In early hematopoietic progenitors, including hematopoietic stem cells (HSCs), multipotent progenitors (MPPs), and at an early stage of the common lymphoid progenitor cells (early CLPs), the two Igκ alleles appear to be identically marked – both are in a relatively inaccessible chromatin state, and are methylated on the DNA. The DNA replicates asynchronously, but the choice of which allele is early replicating switches constantly. At the late CLP stage, the alleles still appear externally similar, but the early replicating allele is now consistently maintained throughout multiple cell divisions. At the pre-B cell stage, leading up to the monoallelic rearrangement event, the late replicating allele relocates to the pericenteromeric heterochromatin, while the early replicating allele moves to a central, euchromatic region of the nucleus. The histones of the early replicating allele become marked with H3Ac and H3K4me3. The RAG protein binds to the H3K4me3 modification. Following DNA demethylation of the early replicating allele, the Igκ locus will undergo monoallelic rearrangement.

The antigen receptor loci replicate in an asynchronous manner. In mature B cells, the rearranged IgH and Igκ alleles replicate early, while the unrearranged alleles replicate in late S phase (24). Although the regions, which replicate asynchronously, are set at implantation, the choice of which of the two alleles will replicate early remains plastic until later stages of development (17, 24). Specifically, in the hematopoietic lineage, the early replicating Igκ allele is not maintained through multiple cell divisions in HSCs and MPPs (Figure 1) (17). This coincides with the fact that at these stages the allele that will undergo rearrangement in pre-B cells has not yet been determined. In the CLP stage, two distinct subgroups are observed. The first subgroup behaves in a manner similar to the HSC and MPP stages, where the replication of each allele switches between early and late timing over the course of numerous cell cycles. In the second CLP subgroup, the identity of the early replicating allele remains constant through multiple cell divisions (Figure 1) (17). This correlates nicely with the fact that the CLP stage contains cells that are not yet committed to the rearrangement of a particular Igκ allele, as well as cells, which will faithfully rearrange only one of the two alleles. It is likely that the allelically committed CLPs represent a more mature stage of differentiation than the allelicaly plastic CLPs. In the pre-B cell stage, the early replicating allele is the one chosen for rearrangement, when the cells are induced to differentiate (Figure 1) (17). Thus, asynchronous replication is seen to be a clear early marker of monoallelic potential, and commitment of a specific allele to replicate early coincides with the commitment of that allele to rearrange later in B cell development. How these patterns are set up, maintained, and translated into monoallelic expression or rearrangement is still unknown and should be the subject of future research.

## Nuclear Localization and Chromatin Structure

An additional level of regulation of the monoallelic choice, which comes into play closer to the actual rearrangement reaction, is that of nuclear localization and chromatin condensation. When genes are localized to the pericenteromeric heterochromatin, they are maintained in a repressive and inaccessible chromatin state, which is not optimal for the activity of the RAG machinery. At the time of recombination of the TCRα (26), TCRβ (27–29), and Igκ (30, 31) loci, it has been observed that one allele is usually located at the nuclear periphery, within a domain of pericenteromeric heterochromatin. The opposing allele is localized to more central, euchromatic regions of the nucleus, or, conversely, is looped away from the nuclear periphery. Only the allele, which is removed from the nuclear periphery, undergoes rearrangement (Figure 1). It is particularly noteworthy that this mechanism is present at the TCRα locus, since allelic exclusion does not occur at this locus (14), yet the rearrangement is restricted to one allele at a time. The ATM protein has been implicated as part of the mechanism that sequesters one of the alleles to the pericentromeric heterochromatin. In the absence of a functional ATM protein, the recruitment to the heterochromatin of Igκ and TCRα is impaired and many cells are observed to have RAG-mediated double strand breaks (DSBs) simultaneously on both alleles (26, 31). This function of ATM is not mediated via its canonical phosphorylation of H2AX or MDC1 as a reaction to DSBs (32). The precise mechanism of action is still not well understood.

Location and condensation are not the only ways in which two homologous antigen receptor alleles differ from each other on the chromatin level prior to rearrangement. In pre-B cells, the Igκ allele, which is destined to undergo rearrangement, is usually packaged with activating histone marks, such as H3K4me3 and H3Ac, whereas the opposing allele, which is associated with the pericenteromeric heterochromatin, is not (Figure 1) (17, 30). The presence of H3K4me3 at the rearrangement site is particularly noteworthy, as this acts as a docking site for the RAG2 protein (33, 34). This protein contains a PHD domain, which specifically recognizes the H3K4me3 modification that is necessary for efficient V(D)J recombination *in vivo* (35–37). Indeed, RAG2 binding at this locus is monoallelic, and occurs on the allele enriched for H3K4me3 (Figure 1) (17). The H3K4me3 mark is present at all of the antigen receptor loci at the developmental stage at which they undergo rearrangement (33) and the RAG complex is specifically recruited to these sites (26, 27, 34). Whether this histone mark is monoallelic at the remaining loci has not yet been examined, so it remains to be seen whether this is a widespread phenomenon.

## DNA Demethylation

We will conclude this review with the discussion of the developmentally regulated removal of methylation from the DNA at the antigen receptor loci, as a final step leading to rearrangement. The antigen receptor loci are methylated in most tissues in the body following the wave of *de novo* DNA methylation, which takes place throughout the genome shortly after implantation (38, 39). It has been observed that following rearrangement in the B cell lineage, the IgH and Igκ loci are hypomethylated on the rearranged allele, while alleles, which are still in the germline configuration, remain hypermethylated (40, 41). Although it is possible that the monoallelic demethylation observed on the rearranged alleles occurs following the recombination process, there are a number of observations, which indicate that the demethylation takes place prior to rearrangement and, in fact, enhances the rearrangement process. For one, DNA methylation has been shown to block the activity of the RAG proteins *in vitro* (42) and reduction of methylation can induce rearrangement in cell culture conditions (43). Demethylation begins on the Igκ locus at the pre-B cell stage (Figure 1), and will occur at this stage even in RAG^−/−^ cells incapable of performing rearrangement (40). In fact, over the course of normal development, demethylation of the Igκ locus will occur in a monoallelic manner, even in the case of feedback inhibition of a prerearranged transgene, which will prevent the rearrangement of the endogenous locus (40, 44). Additionally, in a mouse where both of the endogenous Igκ alleles have been replaced with a prerearranged functional Igκ gene, one of the alleles is unmethylated in mature B cells, while the second remains fully methylated, despite the fact that both alleles are expressed at similar levels (16). Rearrangement intermediates of the Igκ locus (where the DSBs created by the RAG machinery are not yet resolved) are found among the unmethylated, but not methylated, fraction of wild type pre-B cells DNA (44). Moreover, the methylated chromatin fraction from RAG^−/−^ IgH^+^ pre-B cells is not a good substrate for exogenous RAG cleavage, while the unmethylated fraction is (44). Taken together, it is clear that the monoallelic DNA demethylation is a strong mechanism hardwired into B cell development, which is independent of the rearrangement process, but which licenses monoallelic recombination.

A number of *cis* regulatory elements at the Igκ locus contribute to the demethylation process in pre-B cells. These elements include the three κ enhancers (iEκ, 3’Eκ, and Ed) (40, 45–48), as well as the recently characterized Dm element, which lies upstream of iEκ (49). Over the past few years, a number of mechanisms have been suggested, which can lead to demethylation of DNA sequences. Demethylation may occur either in an active manner, which transpires independently of DNA replication, or in a passive manner, where the methylation is not regenerated following replication and is thus diluted over the course of multiple cell divisions. The proteins from the Tet family, which catalyze the oxidation of 5-methyl cytosine (5mC) residues into 5-hydroxymethyl cytosine (5hmC) (50), have been suggested as mediators of DNA demethylation in a number of tissues, in both an active and passive manner. Once the 5hmC intermediate has replaced the 5mC, it can be either be actively excised from the genome and substituted with an unmodified cytosine (51, 52), or, alternatively, may be passively lost over the course of DNA replication (53) [since DNMT1, does not recognize 5hmC as a substrate for maintenance of DNA methylation (54)]. A different strategy of passive demethylation, which has been reported, is the sequestration of DNMT1 from the DNA by *cis* acting non-coding RNA (55). This allows the DNA to become unmethylated by a passive mechanism during cell division. Whether any of the above described mechanisms is utilized by the cell for demethylation of the Igκ locus has yet to be determined.

## Conclusion

Altogether, we see that the cell uses multiple layers of epigenetic regulation, starting from the early post-implantation embryo, to ensure that the antigen receptor genes undergo rearrangement on one allele at a time, and thus allow for the clonal monoallelic expression of the antigen receptors on B and T cells, giving rise to the great diversity and specificity of the system. In the Igκ locus, asynchronous replication is the first epigenetic mark to be fixed upon a specific allele. This is apparently followed by histone modifications, which begin to appear on the chosen allele in the pro-B cell stage, before the alleles move to separate nuclear compartments in pre-B cells (17, 30). In the pre-B cell stage, one allele becomes more strongly marked with active histone modifications, whereas the other allele is located adjacent to the heterochromatin. The final epigenetic change, which precedes rearrangement, is the removal of DNA methylation from the allele that is then cleaved by the recombination machinery. It is not yet clear what the comparative contribution of each epigenetic event is toward the regulation of the monoallelic rearrangement. There is still a large gap in our understanding of how exactly these patterns are established, maintained, and translated into the monoallelic rearrangement phenotype. Future research will improve our understanding of this, and perhaps other monoallelically expressed systems. It will be exciting to see what more can be learned about this fascinating system.

## Conflict of Interest Statement

The authors declare that the research was conducted in the absence of any commercial or financial relationships that could be construed as a potential conflict of interest.
